# Similar TKA designs with differences in clinical outcome: A randomized, controlled trial of 77 knees with a mean follow-up of 6 years

**DOI:** 10.3109/17453674.2011.636677

**Published:** 2011-11-25

**Authors:** Huub J Meijerink, Nico Verdonschot, Corné JM van Loon, Gerjon Hannink, Maarten C de WaalMalefijt

**Affiliations:** ^1^Department of Orthopaedics; ^2^Orthopaedic Research Laboratory, Radboud University, Nijmegen Medical Centre, Nijmegen; ^3^Laboratory for Biomechanical Engineering, University of Twente, Enschede; ^4^Department of Orthopaedics, Rijnstate Hospital, Arnhem, the Netherlands

## Abstract

**Background and purpose:**

To try to improve the outcome of our TKAs, we started to use the CKS prosthesis. However, in a retrospective analysis this design tended to give worse results. We therefore conducted a randomized, controlled trial comparing this CKS prosthesis and our standard PFC prosthesis. Because many randomized studies between different TKA concepts generally fail to show superiority of a particular design, we hypothesized that these seemingly similar designs would not lead to any difference in clinical outcome.

**Patients and methods:**

82 patients (90 knees) were randomly allocated to one or other prosthesis, and 39 CKS prostheses and 38 PFC prostheses could be followed for mean 5.6 years. No patients were lost to follow-up. At each follow-up, patients were evaluated clinically and radiographically, and the KSS, WOMAC, VAS patient satisfaction scores and VAS for pain were recorded.

**Results:**

With total Knee Society score (KSS) as primary endpoint, there was a difference in favor of the PFC group at final follow-up (p = 0.04). Whereas there was one revision in the PFC group, there were 6 revisions in the CKS group (p = 0.1). The survival analysis with any reoperation as endpoint showed better survival in the PFC group (97% (95% CI: 92–100) for the PFC group vs. 79% (95% CI: 66–92) for the CKS group) (p = 0.02).

**Interpretation:**

Our hypothesis that there would be no difference in clinical outcome was rejected in this study. The PFC system showed excellent results that were comparable to those in previous reports. The CKS design had differences that had considerable negative consequences clinically. The relatively poor results have discouraged us from using this design.

Although current results of total knee arthroplasty (TKA) are relatively good, there is still room for improvement. There is constant research and development, with a view to obtaining longer survival rates (Rand et al. 2003, Julin et al. 2010), a better range of motion (high-flex TKA) (McCalden et al. 2009, Choi et al. 2010, Mehin et al. 2010), or a more anatomical reconstruction of the joint—such as posterior and anterior cruciate ligament retaining designs (Ries 2007, Pritchett 2011) and gender-specific TKA (Clarke and Hentz 2008, Kim et al. 2010).

We started to use the CKS prosthesis (Stratec Medical, Oberdorf, Switzerland), based on previous research at our institution showing that the natural patella groove does not have an isolated lateral orientation (Barink et al. 2006). In contrast to our standard prosthesis (PFC; DePuy/Johnson and Johnson, Warsaw, IN) with a lateral orientation of the patellar groove, the trochlea of the CKS prosthesis is deeper and has a neutral direction. However, in a retrospective analysis, after 1 year the CKS prosthesis tended to have worse Knee Society scores (KSSs) (Brokelman et al. 2004). We decided to compare the outcome thoroughly and started a randomized, controlled trial between the CKS and the PFC prostheses.

Many randomized studies of TKAs with different bearings (Harrington et al. 2009, Rahman et al. 2010), cruciate-retaining or -substituting devices (Kim et al. 2009), gender-specific designs (Kim et al. 2010), and high-flex designs (McCalden et al. 2009, Choi et al. 2010, Mehin et al. 2010) generally fail to show superiority of one of the devices over the other. We therefore hypothesized that the seemingly small differences in design between the CKS and PFC system would not lead to differences in clinical outcome in our study.

## Patients and methods

We designed a randomized, controlled trial with 2 posterior cruciate ligament (PCL) retaining total knee designs. The study protocol was approved by the institutional review board at our hospital and it was carried out in line with the Helsinki Declaration. The study was registered in the ClinicalTrials.gov Protocol Registration System (Identifier: NCT 00228137). All patients who were scheduled to undergo primary total knee arthroplasty because of osteoarthritis or rheumatoid arthritis at the Radboud University Nijmegen Medical Centre were considered for inclusion and were enrolled prospectively. Exclusion criteria were dementia, hemophilia, juvenile rheumatoid arthritis, and ligament insufficiency that needed a posterior-stabilized or otherwise more constrained type of design. Between November 2002 and December 2004, 87 consecutive patients (95 knees) were assessed for eligibility. 5 patients (5 knees) were excluded before randomization: 2 patients refused to participate, 2 patients had hemophilia, and 1 patient had dementia.

After written informed consent had been obtained, the knees were randomly allocated to 2 groups. 45 knees received a press-fit condylar prosthesis (PFC; DePuy/Johnson and Johnson, Warsaw, IN) and 45 knees received a continuum knee system prosthesis (CKS; Stratec Medical, Oberdorf, Switzerland). Computer-generated randomization with stratification for age, co-morbidity, and flexion contracture was performed by an independent observer to allocate the patients in equal numbers to the 2 groups.

**Figure 1.**
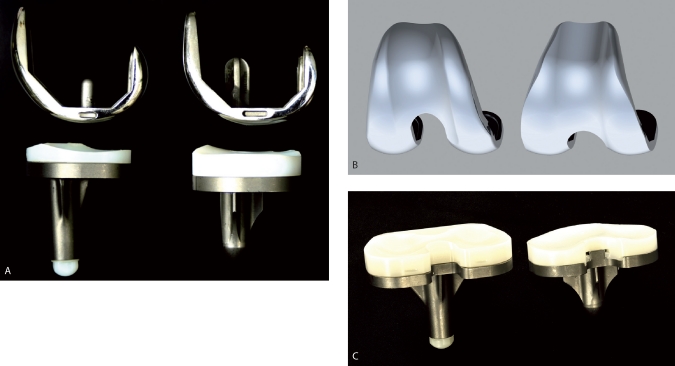
A. Sagittal view of the PFC design (left) and the CKS design (right). B. Anterior view of a computer model of the femoral components. Notice the lateral orientation of the trochlea in PFC (left) and neutral orientation in the CKS component (right). C. Posterior view of the tibial and PE insert components. The central posterior edge of the CKS insert (right) is relatively sharp compared to the PFC insert (left).

Both cemented designs are PCL-retaining and have a fixed polyethelene (PE) insert on a tibial tray with central keel. The femoral and tibial components are made of the same material (cobalt-chromium-molybdenum and titanium-aluminium-vanadium alloy, respectively). In contrast to the lateral orientation of the patellar groove in the PFC prosthesis, the trochlea of the CKS prosthesis is deeper and has a neutral direction. The femoral component of the PFC has a fixation peg in both condyles, whereas the CKS design uses one central peg. Furthermore, the CKS prosthesis has a different surface texture of the femoral component. Additionally, the PE insert of the CKS design has a more prominent and sharp posterior edge compared to the PFC design (Figure 1).

Identical surgical techniques were used in the groups according to the manuals of the designers. 6 surgeons were involved in the study. All procedures were performed by an experienced knee surgeon or under the direct supervision of one. A pneumatic tourniquet was used for all patients. A medial parapatellar capsular incision was used. No patellas were resurfaced. All implants were cemented after pulsed lavage, drying, and pressurization of the cement (Surgical Simplex, Stryker Howmedica). Continuous passive motion was started on the second postoperative day. Thereafter, active range-of-motion exercises and walking were started under the supervision of a physiotherapist.

Routine follow-up evaluation was scheduled at postoperative intervals of 3 months, 6 months, 1 year, and annually thereafter. Preoperative and postoperative review data were recorded by a physician assistant who was blinded regarding patient allocation. At each follow-up visit, we took anteroposterior, lateral, and skyline patellar radiographs, which were evaluated according to the guidelines of the Knee Society (Ewald 1989). The primary endpoint of the study was the between-group difference in total KSS (Insall et al. 1989). Pre-specified secondary endpoints to provide supportive evidence for the primary objective included results on the KSS subscores, the Western Ontario and McMaster Universities Osteoarthritis Index (WOMAC) score (Bellamy et al. 1988), range of motion, survival, and patient satisfaction and pain, both of which were assessed using a visual analog scale (VAS; 0 = total dissatisfaction or no pain and 100 = complete satisfaction or intolerable pain). A reoperation was defined as any operative procedure at the involved knee. A revision was defined as any removal, exchange, or addition of one or more of the prosthetic components.

### Statistics

A sample size estimation showed that 37 knees per group would be required to detect a clinically relevant difference of 10 points with a standard deviation of 15 points in the total KSS, with an alpha of 0.05 and a power of 80%. Intergroup differences were determined using Student's t-test for continuous variables and the Pearson chi-square test or Fisher's exact test for categorical variables. Survival analyses were performed using the Kaplan-Meier method and compared using log-rank tests. Survival estimates are presented with 95% confidence intervals (CIs). For all data sets, differences were considered statistically significant at p-values < 0.05.

## Results

**Figure 2.**
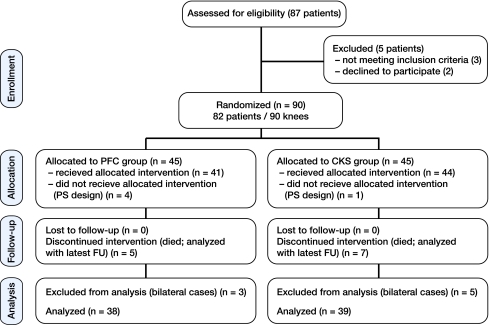
Flow diagram according to the CONSORT guidelines.

After randomization, 5 patients were excluded because a posterior-stabilized design was needed after routinely sacrificing the PCL in cases with a flexion contracture of 25 degrees or more (1 in the CKS group and 4 in the PFC group). Because bilateral involvement might cause bias, 8 other knees were excluded (5 in the CKS group and 3 in the PFC group). No patients were lost to follow-up, but 12 relatively elderly patients died of unrelated causes. These patients were analyzed according to the latest available follow-up. Consequently, we analyzed 39 knees with a CKS prosthesis and 38 knees with a PFC prosthesis (Table 1 and Figure 2), with a mean follow-up of 5.6 (1.2–7.7) years (i.e. 5.4 (1.5–7.7) years for the CKS group and 5.7 (1.2–7.7) years PFC group).

**Table 1.** Patient demographics and baseline clinical status

	PFC group (n = 38)	CKS group (n = 39)
Sex (female/male)	26/12	24/15
Age, years	65 (10; 45–81)	69 (11; 48–88)
Height, cm	169 (9; 154–187)	170 (11; 148–190)
Weight, kg	85 (18; 61–130)	82 (15; 60–120)
BMI	30 (5; 21–45)	28 (4; 21–39)
Diagnosis (OA/RA)	33/5	37/2
ROM, degrees		
Extension	–4 (6; -20 to 0)	–5 (6; -20 to 5)
Flexion	109 (14; 75–135)	111 (19; 70–140)
Total ROM	104 (16; 65–125)	106 (20; 70–140)
KSS, points		
Knee	53 (17; 9–95)	51 (17; 15–91)
Function	37 (20; -5–70)	42 (20; -10–90)
Total	89 (32; 4–150)	92 (29; 40–177)
WOMAC score, points	54 (13; 25–75)	52 (14; 25–95)
VAS pain	62 (17; 26–90)	55 (17; 20–91)

With total KSS as primary endpoint, there was a difference between groups in favor of the PFC group at final follow-up (p = 0.04) (Table 2). Evaluation of the postoperative KSS subscores, WOMAC score, range of motion, VAS for patient satisfaction, and VAS for pain all tended to be superior for the PFC group (Table 2). At final follow-up, there were differences in KSS knee subscore (p = 0.04) and VAS satisfaction (p = 0.04) in favor of the PFC system.

**Table 2.** Clinical results

	PFC group (n=38)	CKS group (n=39)	p-value
Revisions (no.)	1	6	0.1 **^a^**
Reoperations (no.)	1	8	0.03 **^a^**
ROM (deg)			
Extension	-0.8 (4.1; -20–5)	-3.3 (6.9; -20–10)	0.05 **^b^**
Flexion	108 (15; 80–135)	104 (17; 65–140)	0.2 **^b^**
Total ROM	108 (17; 65–135)	100 (21; 45–140]	0.09 **^b^**
KSS (points)			
Knee	88 (12; 59–100)	80 (19; 35–100	0.04 **^b^**
Function	65 (27; -20–100)	55 (30; -10–100	0.1 **^b^**
Total	153 (30; 71–200)	135 (43; 40–200)	0.04 **^b^**
WOMAC score (points)	20 (16; 0–57)	24 (22; 0–79)	0.3 **^b^**
VAS satisfaction	83 (20; 0–100)	71 (27; 0–100)	0.03 **^b^**
VAS pain	17 (24; 0–80)	24 (26; 0–70)	0.3 **^b^**
Radiolucency (no.)	2	3	1 **^a^**

**^a^** Fisher exact test and **^b^** Student t-test.

**Figure 3.**
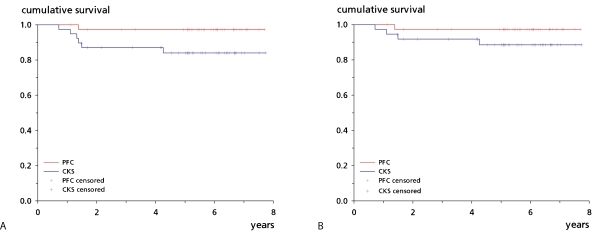
Kaplan-Meier survival plots. A. With revision for any reason as endpoint, the PFC group had a survival of 97% (95% CI: 92–100) after 8 years and the CKS group had a survival of 84% (72–96) (p = 0.05). B. With aseptic revision as endpoint, the PFC group had a survival of 97% (92–100) after 8 years and the CKS group had a survival of 89% (78–99) (p = 0.2).

There was 1 revision in the PFC group; a thicker polyethylene insert was placed for instability. In contrast, there were 6 revisions in the CKS group: in 5 patients, the CKS prosthesis was removed because of poor function and pain and 1 patient was treated with arthrolysis and secondary resurfacing of the patella. During the removal of the prostheses, it appeared that all femoral components of the failed CKS group were easy to remove, leaving an intact cement layer on the bones—indicating inadequate fixation between prosthesis and cement. Cultures were positive in 2 of the CKS revisions. 8-year survival analysis with revision for any reason as endpoint showed 97% (95% CI: 92–100) survival for the PFC group and 84% (72–96) survival for the CKS group (p = 0.05) (Figure 3A). The survival values for aseptic revision were 97% (92–100) and 89% (78–99) respectively (p = 0.2) (Figure 3B).

**Figure 4.**
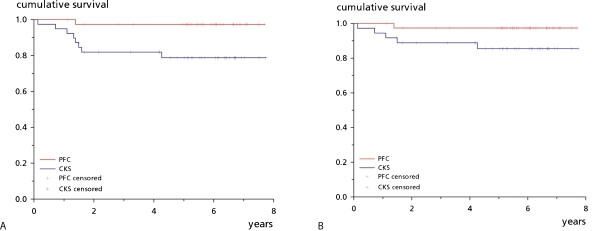
Kaplan-Meier survival plots. A. With any reoperation as endpoint, the PFC group had a survival of 97% (95% CI: 92–100) after 8 years and the CKS group had a survival of 79% (66–92) (p = 0.02). B. With aseptic reoperation as endpoint, the PFC group had a survival of 97% (92–100) after 8 years and the CKS group had a survival of 85% (73–97) (p = 0.08).

2 other patients in the CKS group were reoperated. 1 patient developed postoperative arthrofibrosis and was manipulated under anesthesia, but the knee remained stiff with 20° fixed flexion deformity and 70° of flexion. 1 patient was treated with open debridement followed by antibiotics for 6 months because of a culture-proven deep infection. 4 years later, there were no signs of infection and the knee functioned well. 2 patients (1 in each group) developed a hematoma, both of which were treated conservatively. There were no thromboembolic complications. The 8-year survival analysis with any reoperation as endpoint showed a difference between the PFC group and the CKS group (97% (92–100) for PFC and 79% (CI: 66–92) for CKS; p = 0.02) (Figure 4A). The survival values for aseptic reoperation were 97% (92–100) and 85% (73–97) respectively (p = 0.08) (Figure 4B).

Analysis of the radiographs at final follow-up showed a radiolucency smaller than 2 mm in one zone under the tibial component in 2 cases in the PFC group and in 3 cases in the CKS group. These radiolucent lines were already present in the direct postoperative radiographs and no radiolucent line was progressive; none of these 5 cases were classified as radiographic loosening. The skyline patellar radiographs did not show (sub)luxation of the patella in the PFC group or in the CKS group.

## Discussion

Our hypothesis that there would be no difference in clinical outcome between the PFC prosthesis and the CKS prosthesis was rejected. With total KSS as primary endpoint, and for survival with any reoperation as endpoint, the CKS group showed a worse result. With our standard prosthesis, the PFC, we found an excellent survival rate of 97% for any revision after 8 years. Other authors have recently reported similar 10-year survival rates for the PFC prosthesis: 97% survival for aseptic loosening (Santini and Raut 2008) and 97% survival for revision with any reason other than infection as endpoint (Dalury et al. 2008). The functional results of the PFC prosthesis in our study were also comparable to those in previous reports (Dalury et al. 2008, Harrington et al. 2009, Hanusch et al. 2010).

The excellent clinical scores of the PFC prosthesis do not leave a lot of room for improvement, which is probably why recent RCTs have failed to show a superior design (Harrington et al. 2009, Choi et al. 2010, Rahman et al. 2010). Subtle differences in outcome after TKA require more sensitive instruments. It has been reported that patient-based questionnaires such as WOMAC and the KSS are subjective and largely influenced by pain (Terwee et al. 2006, Stratford and Kennedy 2006). Objective, functional tests may be a valuable additional tool in comparing TKA systems. We have previously shown that monitoring of both knee extension velocity and loading symmetry during sit-to-stand movements is objective and has good discriminative capacity (Boonstra et al. 2008). Similar performance-based measurements to quantify functionality in TKA patients have been reported by others (Podsiadlo and Richardson 1991, Su et al. 1998, Mizner and Snyder-Mackler 2005).

In addition, we have to realize that the outcome after TKA not only depends on the type of implant; Fortin et al. (1999, 2002) stated that the preoperative status of the patient is the strongest determinant of functional outcomes after hip and knee surgery, and Noble et al. (2006) and Mahomed et al. (2002) emphasized the importance of the expectations of the patients. Nevertheless, our study showed an inferior outcome with the CKS design. Although different results have been published about the CKS prosthesis in the limited amount of literature that is available (Martucci et al. 1996, Gobel and Schultz 2008), a 79% survival rate for any reoperation after 8 years in our study is unacceptably low—which made us decide to stop further using the CKS implant system.

The question remains as to why we found such a difference between the PFC and the CKS prostheses, as the designs appear to be quite similar. Concerning the articular part of the prosthesis, the most prominent difference is the orientation of the patella groove. Although patellofemoral complaints are one of the complications after TKA, with the highest incidence (1–24%) and an important reason for revision surgery (Boyd et al. 1993, Healy et al. 1995, Harwin 1998), it seems to be illogical that only a more anatomical trochlea orientation in the CKS design would be responsible for a worse outcome.

An important issue is the observation of bad fixation strength of the prosthetic components to the bone. It appeared that all femoral components of the failed CKS group were easy to remove. Only 2 revisions could be attributed to positive bacterial cultures; the other 3 revisions were defined as aseptic loosening after 13, 16, and 51 months. This high rate of aseptic loosening is uncommon, especially at this early stage (Bozic et al. 2005, Vessely et al. 2006, Schwartz et al. 2010). Moreover, during the removal of the CKS prostheses there was an intact cement layer on the bones, indicating inadequate fixation between the prosthesis and cement. Thus, we believe that an important problem of the CKS design is limited cement-metal interfacial strength of the femoral components.

We therefore wondered what the reason could be for a weak cement-metal interface of the CKS components. We analyzed the differences in the backside of both designs. The femoral component of the PFC has fixation pegs in both condyles, whereas the CKS design uses only 1 central peg. 2 pegs might enhance the fixation relative to 1 central peg, but to our knowledge this has not been described in the literature. We also analyzed the surface roughness of the femoral components and found that the CKS components had a lower surface roughness value than the PFC design (Ra = 1.3 ± 0.1 µm vs. 1.9 ± 0.3µm; p = 0.01). As shown by Walsch et al. (2004) and Manley et al. (1985), a lower surface roughness reduces fixation strength of the implant-cement interface and may explain why the revised CKS components could be removed so easily.

Another difference between the designs concerns the PE insert. The CKS design uses a tibial insert with a more prominent and sharp posterior edge compared to the PFC design. This sharp edge may come in contact with the PCL, leading to damage and subsequent PCL laxity. In a previous study comparing the CKS and the PFC prostheses, kinematic analysis supported the suspicion that the CKS design has larger AP translations than the PFC design (Ploegmakers et al. 2010). Although clinical ratings such as the KSS, total WOMAC, and VAS did not show any statistically significant difference in that study, subscores regarding higher flexion and higher-demanding activities showed greater limitations in knees with a CKS design. Moreover, it has been described that in addition to AP instability, PCL insufficiency may cause (anterior knee) pain and result in malfunction (Waslewski et al. 1998, Pagnano et al. 1998). Thus, the worse functional outcome for the CKS system that we found may also be explained by PCL insufficiency due to in vivo damage of the PCL at the sharp posterior edge of the tibial insert.

Our study had some limitations. First, a relatively high number of patients (12) died before final follow-up. Even so, all the patients were analyzed with the latest available (and minimal 1-year) follow-up. Including these patients, the mean follow-up was 5.6 years. Furthermore, no patients were lost to follow-up. Another possible limitation is the potential bias from there being 6 different surgeons involved in this study. However, since we are a teaching hospital all procedures were performed by—or under direct supervision of—an experienced knee surgeon and none of the reoperated cases had originally been operated by a surgeon with low volume.

One strength of our study was the randomization process with stratification for age, flexion contracture, and co-morbidity. Consequently, patient demographics and the baseline clinical status of both groups were similar. Thus, we are convinced that the differences in clinical function and survival between the groups were caused by the differences in design between the CKS and the PFC prostheses. Our study was not designed to determine the reason for the worse results of the CKS design. We believe that the reason may have been multi-factorial, and a combination of low fixation strength and possible PCL insufficiency. Initially, we thought that the CKS system was very similar to the PFC system, but the large differences in clinical outcome were evident and discouraged us from further use of the CKS system.

## References

[CIT0001] Barink M, Van de Groes S, Verdonschot N, De Waal Malefijt M (2006). The difference in trochlear orientation between the natural knee and current prosthetic knee designs; towards a truly physiological prosthetic groove orientation. J Biomech.

[CIT0002] Bellamy N, Buchanan WW, Goldsmith CH, Campbell J, Stitt LW (1988). Validation study of WOMAC: a health status instrument for measuring clinically important patient relevant outcomes to antirheumatic drug therapy in patients with osteoarthritis of the hip or knee. J Rheumatol.

[CIT0003] Boonstra MC, de Waal Malefijt MC, Verdonschot N (2008). How to quantify knee function after total knee arthroplasty?. Knee.

[CIT0004] Boyd AD, Ewald FC, Thomas WH, Poss R, Sledge CB (1993). Long-term complications after total knee arthroplasty with or without resurfacing of the patella. J Bone Joint Surg (Am).

[CIT0005] Bozic KJ, Kinder J, Meneghini RM, Zurakowski D, Rosenberg AG, Galante JO (2005). Implant survivorship and complication rates after total knee arthroplasty with a third-generation cemented system: 5 to 8 years followup. Clin Orthop.

[CIT0006] Brokelman RB, Meijerink HJ, de Boer CL, van Loon CJ, de Waal Malefijt MC, van Kamapen A (2004). Are surgeons equally satisfied after total knee arthroplasty?. Arch Orthop Trauma Surg.

[CIT0007] Choi WC, Lee S, Seong SC, Jung JH, Lee MC (2010). Comparison between standard and high-flexion posterior-stabilized rotating-platform mobile-bearing total knee arthroplasties: a randomized controlled study. J Bone Joint Surg (Am).

[CIT0008] Clarke HD, Hentz JG (2008). Restoration of femoral anatomy in TKA with unisex and gender-specific components. Clin Orthop.

[CIT0009] Dalury DF, Barrett WP, Mason JB, Goldstein WM, Murphy JA, Roche MW (2008). Midterm survival of a contemporary modular total knee replacement: a multicentre study of 1970 knees. J Bone Joint Surg (Br).

[CIT0010] Ewald FC (1989). The Knee Society total knee arthroplasty roentgenographic evaluation and scoring system. Clin Orthop.

[CIT0011] Fortin PR, Clarke AE, Joseph L, Liang MH, Tanzer M, Ferland D, Phillips C, Partridge AJ, Belisle P, Fossel AH, Mahomed N, Sledge CB, Katz JN (1999). Outcomes of total hip and knee replacement: preoperative functional status predicts outcomes at six months after surgery. Arthritis Rheum.

[CIT0012] Fortin PR, Penrod JR, Clarke AE, St-Pierre Y, Joseph L, Belisle P, Liang MH, Ferland D, Phillips CB, Mahomed N, Tanzer M, Sledge C, Fossel AH, Katz JN (2002). Timing of total joint replacement affects clinical outcomes among patients with osteoarthritis of the hip or knee. Arthritis Rheum.

[CIT0013] Gobel D, Schultz W (2008). Clinical results and economics of two primary total knee replacement systems implanted in standardised surgical technique. Z Orthop Unfall.

[CIT0014] Hanusch B, Lou TN, Warriner G, Hui A, Gregg P (2010). Functional outcome of PFC Sigma fixed and rotating-platform total knee arthroplasty. A prospective randomised controlled trial. Int Orthop.

[CIT0015] Harrington MA, Hopkinson WJ, Hsu P, Manion L (2009). Fixed- vs mobile-bearing total knee arthroplasty: does it make a difference?--a prospective randomized study. J Arthroplasty.

[CIT0016] Harwin SF (1998). Patellofemoral complications in symmetrical total knee arthroplasty. J Arthroplasty.

[CIT0017] Healy WL, Wasilewski SA, Takei R, Oberlander M (1995). Patellofemoral complications following total knee arthroplasty. Correlation with implant design and patient risk factors. J Arthroplasty.

[CIT0018] Insall JN, Dorr LD, Scott RD, Scott WN (1989). Rationale of the Knee Society clinical rating system. Clin Orthop.

[CIT0019] Julin J, Jamsen E, Puolakka T, Konttinen YT, Moilanen T (2010). Younger age increases the risk of early prosthesis failure following primary total knee replacement for osteoarthritis. A follow-up study of 32,019 total knee replacements in the Finnish Arthroplasty Register. Acta Orthop.

[CIT0020] Kim YH, Choi Y, Kwon OR, Kim JS (2009). Functional outcome and range of motion of high-flexion posterior cruciate-retaining and high-flexion posterior cruciate-substituting total knee prostheses. A prospective, randomized study. J Bone Joint Surg (Am).

[CIT0021] Kim YH, Choi Y, Kim JS (2010). Comparison of a standard and a gender-specific posterior cruciate-substituting high-flexion knee prosthesis: a prospective, randomized, short-term outcome study. J Bone Joint Surg (Am).

[CIT0022] Mahomed NN, Liang MH, Cook EF, Daltroy LH, Fortin PR, Fossel AH, Katz JN (2002). The importance of patient expectations in predicting functional outcomes after total joint arthroplasty. J Rheumatol.

[CIT0023] Manley MT, Stern LS, Gurtowski J (1985). The load carrying and fatigue properties of the stem-cement interface with smooth and porous coated femoral components. J Biomed Mater Res.

[CIT0024] Martucci E, Verni E, Del PG, Stulberg SD (1996). CKS knee prosthesis: biomechanics and clinical results in 42 cases. Chir Organi Mov.

[CIT0025] McCalden RW, Macdonald SJ, Bourne RB, Marr JT (2009). A randomized controlled trial comparing “high-flex” vs “standard” posterior cruciate substituting polyethylene tibial inserts in total knee arthroplasty. J Arthroplasty.

[CIT0026] Mehin R, Burnett RS, Brasher PM (2010). Does the new generation of high-flex knee prostheses improve the post-operative range of movement?: a meta-analysis. J Bone Joint Surg (Br).

[CIT0027] Mizner RL, Snyder-Mackler L (2005). Altered loading during walking and sit-to-stand is affected by quadriceps weakness after total knee arthroplasty. J Orthop Res.

[CIT0028] Noble PC, Conditt MA, Cook KF, Mathis KB (2006). The John Insall Award: Patient expectations affect satisfaction with total knee arthroplasty. Clin Orthop.

[CIT0029] Pagnano MW, Hanssen AD, Lewallen DG, Stuart MJ (1998). Flexion instability after primary posterior cruciate retaining total knee arthroplasty. Clin Orthop.

[CIT0030] Ploegmakers MJ, Ginsel B, Meijerink HJ, de Rooy JW, de Waal Malefijt MC, Verdonschot N, Banks SA (2010). Physical examination and in vivo kinematics in two posterior cruciate ligament retaining total knee arthroplasty designs. Knee.

[CIT0031] Podsiadlo D, Richardson S (1991). The timed “Up & Go”: a test of basic functional mobility for frail elderly persons. J Am Geriatr Soc.

[CIT0032] Pritchett JW (2011). Patients prefer a bicruciate-retaining or the medial pivot total knee prosthesis. J Arthroplasty.

[CIT0033] Rahman WA, Garbuz DS, Masri BA (2010). Randomized controlled trial of radiographic and patient-assessed outcomes following fixed versus rotating platform total knee arthroplasty. J Arthroplasty.

[CIT0034] Rand JA, Trousdale RT, Ilstrup DM, Harmsen WS (2003). Factors affecting the durability of primary total knee prostheses. J Bone Joint Surg (Am).

[CIT0035] Ries MD (2007). Effect of ACL sacrifice, retention, or substitution on kinematics after TKA. Orthopedics.

[CIT0036] Santini AJ, Raut V (2008). Ten-year survival analysis of the PFC total knee arthroplasty–a surgeon's first 99 replacements. Int Orthop.

[CIT0037] Schwartz AJ, la Valle CJ, Rosenberg AG, Jacobs JJ, Berger RA, Galante JO (2010). Cruciate-retaining TKA using a third-generation system with a four-pegged tibial component: a minimum 10-year followup note. Clin Orthop.

[CIT0038] Stratford PW, Kennedy DM (2006). Performance measures were necessary to obtain a complete picture of osteoarthritic patients. J Clin Epidemiol.

[CIT0039] Su FC, Lai KA, Hong WH (1998). Rising from chair after total knee arthroplasty. Clin Biomech (Bristol, Avon ).

[CIT0040] Terwee CB, van der Slikke RM, van Lummel RC, Benink RJ, Meijers WG, de Vet HC (2006). Self-reported physical functioning was more influenced by pain than performance-based physical functioning in knee-osteoarthritis patients. J Clin Epidemiol.

[CIT0041] Vessely MB, Whaley AL, Harmsen WS, Schleck CD, Berry DJ (2006). The Chitranjan Ranawat Award: Long-term survivorship and failure modes of 1000 cemented condylar total knee arthroplasties. Clin Orthop.

[CIT0042] Walsh WR, Svehla MJ, Russell J, Saito M, Nakashima T, Gillies RM, Bruce W, Hori R (2004). Cemented fixation with PMMA or Bis-GMA resin hydroxyapatite cement: effect of implant surface roughness. Biomaterials.

[CIT0043] Waslewski GL, Marson BM, Benjamin JB (1998). Early, incapacitating instability of posterior cruciate ligament-retaining total knee arthroplasty. J Arthroplasty.

